# Safety of Mealworm Meal in Layer Diets and their Influence on Gut Morphology

**DOI:** 10.3390/ani11051439

**Published:** 2021-05-18

**Authors:** Ondrej Stastnik, Jakub Novotny, Andrea Roztocilova, Petr Kouril, Vojtech Kumbar, Julius Cernik, Libor Kalhotka, Leos Pavlata, Lubor Lacina, Eva Mrkvicova

**Affiliations:** 1 Department of Animal Nutrition and Forage Production, Faculty of AgriSciences, Mendel University in Brno, Zemědělská 1, 613 00 Brno, Czech Republic; jakub.novotny@mendelu.cz (J.N.); andrea.roztocilova@mendelu.cz (A.R.); leos.pavlata@mendelu.cz (L.P.); eva.mrkvicova@mendelu.cz (E.M.); 2 Department of Agrochemistry, Soil Science, Microbiology and Plant Nutrition, Faculty of AgriSciences, Mendel University in Brno, Zemědělská 1, 613 00 Brno, Czech Republic; petr.kouril@mendelu.cz (P.K.); libor.kalhotka@mendelu.cz (L.K.); 3 Department of Technology and Automobile Transport (Section Physics), Faculty of AgriSciences, Mendel University in Brno, Zemědělská 1, 613 00 Brno, Czech Republic; vojtech.kumbar@mendelu.cz; 4 Department of Pathological Morphology and Parasitology, Faculty of Veterinary Medicine, University of Veterinary Sciences Brno, Palackého Třída 1946/1, 612 42 Brno, Czech Republic; cernikj@vfu.cz; 5 Underground Food s.r.o., Brno, Zeiberlichova 160/72, 644 00 Brno, Czech Republic; lacina@ugf.cz

**Keywords:** *Tenebrio molitor*, poultry nutrition, insect, villus, yellow mealworm, hen’s, viscosity, feed microbiology

## Abstract

**Simple Summary:**

There is limited research on the use of the mealworm meal in laying hens’ diets and effects on relative organ weights, caecum microbiota, ileum morphology and digesta viscosity. All these parameters can affect the performance of animals, i.e., the laying and quality of eggs. The mealworm meal is a relatively new feedstuff, where it is necessary to exclude a possible harmful effect. Insect products have a beneficial nutrient content, but there are issues of stability, shelf life, storage and contamination, which could, in the case of negative properties, affect the morphology of the digestive tract, cause liver damage and, as a result, affect the animal performance parameters. The main objective of this study was to verify the safety of the mealworm meal in the feed of laying hens from 17–42 weeks of age. Therefore, the feed mixtures were tested in terms of microbiological stability, fungal and mycotoxin content and selected parameters of hens’ intestinal morphology and physiology were monitored. Feed mixtures with proportions of insect products were microbially stable even after four months. Based on the results of this study, use of two to five percent of mealworm meal in hen′s diet may be used as a sustainable and safe protein feed.

**Abstract:**

The main objective of this study was to verify the safety of mealworm meal in the feed of laying hens from 17 to 42 weeks of age. Therefore, the feed mixtures were tested in terms of microbiological stability, fungal and mycotoxin content and selected parameters of hens’ intestinal morphology and physiology were monitored. The experiment was carried out with 30 Lohmann Brown Classic hens. Hens were divided by body mass into three equal groups with 10 replicates per treatment. The two experimental groups received feed mixtures containing 2% and 5% yellow mealworm (*Tenebrio molitor* L.) meal. The third group was a control group which had 0% of mealworm meal in the diet. Diets with 2% and 5% of mealworm meals did not affect the length of villi and microbiome of the caecum. The highest digesta viscosity from the ileum was found in the group with 5% mealworm, which may indicate a slower passage of the digesta through the digestive tract. Based on our results, it may be concluded that the proportion of mealworm meals does not deteriorate the quality of feeds. Mealworm meal does not negatively affect microbial stability in experimental feeds. Therefore, it can be recommended the two and (or) five percent of mealworm meal inclusion in hen’s diet.

## 1. Introduction

Given the fact that the growth of the human population is expected, the need to increase the production of animal products is proportionally unavoidable [1]. Poultry production is one of the cheapest and easiest ways to obtain a supply of animal protein for fast egg and meat production and beneficial feed conversion efficiency [2]. Thus, eggs represent a low-cost source of high-quality protein, lipids, vitamins, minerals, low in calories with the content of various amino acids [3]. With regard to this, egg production has been increasing rapidly, expected to reach 89 million tons by 2030 [4]. It is desirable to include readily available, low-cost feedstuffs in the diet of laying hens, which may provide the nutrients for optimizing egg production. Poultry production depends heavily on plant protein sources such as soybean (extracted) meal. However, the rising price of feedstuffs and potential shortages of ingredients in the future [5] due to adverse climatic conditions, land unavailability, and overexploitation of marine sources could lead to serious consequences regarding feed and food production, and the situation will be further worsened by the food–feed–fuel competition [6]. Moreover, proteins from insect products could become a source of animal protein for laying hens and thus ensure a quality nutrient composition of eggs [7]. In this respect, insects appear to be a suitable alternative feed (or food), which is considered to be of animal origin. Generally, insects have proven to be a good alternative feedstuff, especially for poultry, because insects are a part of the natural poultry diet [8,9]. It is currently being investigated whether insect products as a feedstuff may affect the microbial population of the digestive tract in animals.

It is well-known that optimal gastrointestinal health and functionality is important for sustainable animal performance since it has direct effects on animal health and production [10]. It has been reported that the most predominant genera found in chicken cecum are *Clostridium, Ruminococcus, Lactobacillus, Bacteroides* [11,12,13,14,15] and in a smaller representation *Alistipes* and *Faecalibacterium* [11]. For example, it was evaluated the in vitro antimicrobial activities of two fats from *Hermetia illucens* and *Tenebrio molitor* and their effect as a total substitute for dietary soybean oil in cecal fermentation and gut microbiota of growing rabbits. The in vitro activities of *Hermetia illucens* or *Tenebrio molitor* fats against *Pasteurella, Yersinia*, and known pathogens of the rabbit gut, indicate a potential for impairing their growth in vivo in rabbits. The inclusion of *Hermetia illucens* and *Tenebrio molitor* fats in rabbits’ diet stimulated volatile fatty acids production at caecum and could positively modulate the caecal and fecal microbiota of the growing rabbits [16]. Morphometric measurements of villus height and crypt depth are usually used to evaluate intestinal development [17], since they represent useful indicators of gut proliferative and absorptive compartments [18].

The inclusion of 10–15% of mealworm meal into the broiler chickens’ diet could negatively affect cecal microbiota and intestinal mucin dynamics. Therefore, it is recommended to include 5% of *Tenebrio molitor* (TM) meal into broilers’ diet. The authors stated that yellow mealworm meal utilization at low inclusion rates (5%) represents the most feasible alternative in terms of gut microbiota characteristics and mucin dynamics in broiler chickens [19]. In another study, it was noted that TM could be successfully used to replace 4% of extracted soybean meal in laying hens’ diet [20].

There is limited research on the use of the mealworm meal in laying hens’ diets and effects on relative organ weights, caecum microbiota, ileum morphology and digesta viscosity. All these parameters can affect the performance of animals, i.e., the laying and quality of eggs. The mealworm meal is a relatively new feedstuff, where it is necessary to exclude a possibly harmful effect. Insect products have a beneficial nutrient content, but there are issues of stability, shelf life, storage and contamination, which could, in case of negative properties, affect the morphology of the digestive tract, cause liver damage and, as a result, affect the animal performance parameters. The main objective of this study was to verify the safety of the mealworm meal in the feed of laying hens from 17–42 weeks of age. Therefore, the feed mixtures were tested in terms of microbiological stability, fungal and mycotoxin content and selected parameters of hens’ intestinal morphology and physiology were monitored.

## 2. Materials and Methods

The animal procedures were reviewed and approved by the Animal Care Committee of Mendel University in Brno and by the Ministry of Education, Youth and Sports (MSMT-22771/2019-5).

### 2.1. Animals and Diets

The experiment was carried out with 30 Lohmann Brown Classic hens. Layers were housed in balance cages and divided by body mass into three equal groups with 10 replicates per treatment. The two experimental groups received feed mixtures containing 2% (TM2; *n* = 10) and 5% (TM5; *n* = 10) of yellow mealworm (*Tenebrio molitor* L.) meal. The third group (TM0; *n* = 10) was a control group that had 0% of mealworm meal in the diet. The mealworm meal was mixed with the other components into homogeneous feed mixtures. The mealworm meal replaced the appropriate proportion of soybean extracted meal. The yellow mealworm meal was obtained from Underground Food, s.r.o. (Brno, Czech Republic).

From 17–18 weeks of hens’ age, a preparatory period was carried out. Hens were fed with experimental and control diets in this period. The experiment was carried out from 18 to 42 weeks of age. Table 1 shows the composition and proximate analyses of the diets. The rations were calculated according to the Lohmann Tierzucht, Management Guide [21] as isonitrogenous and isoenergic ones. The mash form diets were offered to hens. The hens had free access to feed and water. The health status was evaluated daily.

The chemical composition of the mealworm meal and diets were determined for dry matter, crude protein, ether extract, crude fiber and ash according to Commission regulation (EC) 152/2009 [22]. Room temperature, humidity and lighting regime were controlled according to the requirement for the current age of hens [21].

### 2.2. Measurement of Digestive Tract and Ileum Viscosity

In the 42nd week of age, seven average hens from each group were selected and slaughtered. The entire digestive tracts were removed and divided into the following sections: crop, proventriculus, gizzard, duodenum, jejunum, ileum, caeca and colon. The lengths (or width) and empty weights of each segment were recorded. The fresh cecal contents were transferred to a vial and kept refrigerated for subsequent microbiological analysis.

The fresh digesta (from each hen) was removed from the distal part of the ileum to determine viscosity according to Yasar [23]. The digesta was collected in tubes and then centrifuged for 10 min at 3000 rpm. The resulting supernatant was pipetted into Eppendorf tubes. The samples were analyzed for dynamic viscosity on an RST rheometer (Brookfield, MA, USA) at a constant shear strain rate of 50 s^−1^ with a standard cone-plate geometric arrangement (RCT-50-2; α = 2°), including a temperature duplicator system. The measurement was performed in 10 replicates at 40 °C and the sample volume was 1.2 mL.

### 2.3. Histo-Morphological Measurements

#### 2.3.1. Histopathological Examination

Liver and ileum samples (seven replications in each group) fixed in 10% formalin were treated with a conventional paraffin method, stained with hematoxylin-eosin [24,25] and evaluated under a light microscope (Motic BA 310).

#### 2.3.2. Morphometric Examination

Histopathological preparations stained with hematoxylin-eosin were further used for morphometric examination by the method of image computer analysis Soft Imaging System Cell F—Imaging Software for Life Science Microscopy, OLYMPUS Soft Imaging Solutions. The method of the traditional and automatic computer morphometry in 2D projection was used to measure the length of villi. Morphometric (histometric) measurements were performed based on an image obtained from a classical histological specimen (slide) viewed with a light microscope (Olympus BX 51 with Olympus DP 70 scanner), which is connected to a computer. The display resolution (1360 × 1024) was set in the image computer analysis program mentioned above, the analysis object was displayed on the computer monitor screen with a built-in video camera, and an area in the specimen (ROI) was selected in which the measurement objects (villi) were displayed most suitably. The area was stopped, the most suitable magnification for the given measurement was selected and the analyzed objects were focused and then photographed with a built-in digital camera. The “Arbitrary line” (Al) function was selected on the menu bar for morphometry (any distance—the shortest distance between the selected fixed starting point and the final, variable, measured dimension point—it can be said that the measurement took place in the longitudinal axes of structures) and, using this function, the length of the villi was measured. The length of 3 villi was measured in each section (three sections were made), each from the base to the apex.

### 2.4. Microbiologically Analysis

Microbiological analysis was performed by standard plate methods. Firstly, the mealworm meal and other feedstuffs (wheat, maize, soybean meal) were analyzed microbiologically. Secondly, final feed mixtures were analyzed to determine the quantities of microorganisms.

A 10-g sample of the feed mixture was weighed into a sterile Erlenmeyer flask and 90 mL of sterile saline was added. Each sample was prepared in two duplicates, were the plates done in duplicate for each used dilution to improve accuracy. The samples were homogenized on the orbital shaker PSU-10i (Biosan, Latvia) for 10 min. The following groups of microorganisms were determined by standard procedures. The following constituents were determined in the feed mixture: total plate count on PCA (Biokar Diagnostics, Allonne, France) at 30 °C for 72 h, *E. coli* and other coliforms on Rapid *E. coli* Agar (Bio Rad, Helsinki, Finland) at 37 °C for 24 h, micromycetes (yeasts and moulds) on Chloramphenicol Glucose Yeast Extract Agar (Biokar Diagnostics, Allonne, France) at 25 °C for 120 h, Enterococci on Compass *Enterococcus* agar (Merck, Darmstadt, Germany) at 42 °C for 24 h, *Clostridium perfringens* on TSN Agar (Biokar Diagnostics, Allonne, France) at 45 °C for 24 h, lactic acid bacteria on MRS agar (Biokar Diagnostics, Allonne, France) at 30 °C for 72 h, *Staphylococcus aureus* on Baird-Parker agar with rabbit plasma (Biokar Diagnostics, Allonne, France) at 37 °C for 24 h and *Salmonella* spp. by double enrichment method on Rapid Salmonella Agar (Bio Rad, Helsinki, Finland) at 37 °C for 24 h.

From the obtained samples of cecal chyme (*n* = 7 per group), 5 g were collected and placed into a sterile centrifuge tube, 45 mL of sterile saline solution were added and the mixture was shaken for 1 min on Multi-speed Vortex MSV-3500 (Biosan, Latvia). The following constituents were determined in cecal chyme: *E. coli* and other coliform bacteria on Rapid *E. coli* Agar (Bio Rad, Helsinki, Finland) at 37 °C for 24 h, *Clostridium perfringens* on TSN Agar (Biokar Diagnostics, Allonne, France) and *Salmonella* spp. by the double-enrichment method on Rapid Salmonella Agar (Bio Rad, Helsinki, Finland) at 37 °C for 24 h. After the incubation, the number of typical colonies was counted and the results were expressed in log CFU/g.

### 2.5. Preparation of Samples for the Analyses of Mycotoxins by HPLC

Mycotoxin analyses were performed at Grain Quality Laboratory, Agrotest Fyto Ltd. (Kroměříž, Czech Republic) by means of high-performance liquid chromatograph Shimadzu LC-20AD equipped with UV (SPD-M20A) and fluorescence (RF-10AXL) detectors (Shimadzu, Kyoto, Japan). Mealworm larvae meal and feed mixtures were analyzed for ochratoxin A (OTA), aflatoxins (AF) (aflatoxin B1 (AFB1) and sum of aflatoxins B1, B2, G1, G2), and deoxynivalenol (DON). Samples were milled using a laboratory mill (Pulverisette 19, Fritsch, Idar-Oberstein, Germany) with a 1-mm screen. For sample preparation, MycoSepâ cleanup columns (Romer Labs Diagnostic GmbH, Tulln, Austria) were used, an appropriate type for the respective mycotoxin, and instructions of the manufacturer were followed.

The method for DON analysis was based on the standard method for trichothecene analysis [26]. In brief, the homogenous sample (12.5 g) was extracted with acetonitrile and water (84:16). The mixture was shaken intensively for 30 min, filtered through Whatman No.1 filter paper and 8 mL volume of the supernatant was passed through a MycoSepâ 227 Trich+ cleanup column. The sample was dried with nitrogen gas and reconstituted in a mobile phase (acetonitrile and water; 90:10). The final sample was injected into the HPLC system, the HPLC conditions were as follows—mobile flow rate 1.0 mL/min, temperature 35 °C, injection rate 25 μL, UV detector (218 nm).

For OTA analyses, the homogenous sample (12.5 g) was extracted with acetonitrile and water (84:16). The mixture was shaken intensively for 30 min, filtered through Whatman No.113V filter paper, and after the addition of glacial acetic acid to the supernatant, the mixture was passed through a MycoSepâ 229 Ochra cleanup cartridge. The sample was dried with nitrogen gas and reconstituted with mobile phase (water and acetonitrile and acetic acid, 60:40:1). The HPLC conditions were as follows—mobile flow rate 0.3 mL/min, temperature 30 °C, injection rate 25 μL, fluorescence detector (460 and 333 nm).

For aflatoxin analyses, the ground test sample (12.5 g) was extracted with methanol and water (80:20). The mixture was shaken for 30 min, filtered through Whatman No.1 filter paper, and mixed with acetonitrile (1:1), the mixture was passed through a MycoSepâ 226 AflaZon+ cleanup column. The sample was diluted (1:3) in the mobile phase (water and methanol, 55:45) and used for HPLC analyses. The HPLC conditions were as follows—mobile flow rate 0.8 mL/min, temperature 25 °C, injection rate 100 μL, fluorescence detector (440 and 360 nm).

Chemicals were of HPLC grade and they were purchased together with analytical standards from Merck (Merck-Sigma-Aldrich s.r.o., Prague, Czech Republic).

Mycotoxins in mealworms larvae meal and feed mixtures were analyzed in duplicates. The detection limits were 40 μg/kg, 0.2 μg/kg and 0.1 μg/kg for DON, OTA and aflatoxins, respectively.

### 2.6. Statistical Analysis

The data were processed by Microsoft Excel (USA) and StatSoft Statistica version 12.0 (USA). The *Shapiro-Wilk W* test was used to test the normality of the data distribution. The data set was well-modeled by a normal distribution. A one-way analysis of variance (ANOVA) was used to determine the differences between the groups. To ensure evidential differences, Scheffé’s test was applied and *p* < 0.05 was regarded as a statistically significant difference.

## 3. Results

The used mealworm meal contained in the dry matter basis 532.5 g/kg of crude protein, 293.5 g/kg of ether extract, 62.1 g/kg of crude fiber and 39.0 g/kg of crude ash.

**Figure 1:**
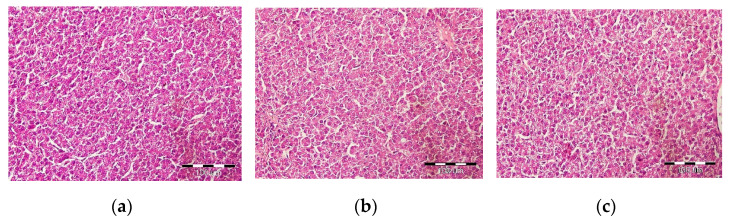
Histopathological examination of hens’ liver: (**a**) TM0—hens receiving control diet; (**b**) TM2—hens receiving a diet with 2% of mealworm meal; (**c**) TM5—hens receiving a diet with a proportion of 5% of mealworm meal.

The results are presented in Table 2, Table 3 and Table 4 and Figure 1. Table 2 brings relative sizes of individual sections of the digestive tract. The morphology of the ileum and the viscosity of the Ileal digesta are shown in Table 3. Figure 1 shows histopathological examination of the liver. Table 4 shows the results of caeca microbial colonization.

### 3.1. Measurements of Digestive Tract

Table 2 shows the mean weights and lengths of individual sections of the digestive tract of hens. In the control group of hens, statistically significant (*p* < 0.05) lower width and height of the gizzard were found. The highest gizzard muscle height was found in the control group compared to the TM2 group. A significantly (*p* < 0.05) longer colon was found in the control group compared to the group of hens receiving a 5% proportion of mealworms in the diet.

### 3.2. Histo-Morphological Examination and Digesta Viscosity

The morphology of the ileum and the viscosity of the ileum digesta were also measured in the experiment. No statistically significant differences were found in the length of villi between the groups (*p* > 0.05). See Table 3. A statistically significant (*p* < 0.05) lowest difference in digesta viscosity was found in the control group compared to the TM5 group.

In the examined ileal samples of the TM0 group, the sites of vacuolation of the cytoplasm of epithelial cells and in the lamina propriae mucosae were detected. In the TM2 group, the sites of vacuolation of the cytoplasm of epithelial cells were detected. In the TM5 group, suspected lymphodepletion was found in some areas of the lymphatic tissue. Otherwise, the samples were without pathological changes.

In the examined liver samples of the TM0 group (*n* = 7), small-capsule vacuolation of the cytoplasm (probably fat capsules), rather periportally, was found in some hepatocytes. The nuclei remain in the center of hepatocytes in many cells. In TM2 samples (*n* = 7), some hepatocytes had small-droplet cytoplasmic vacuolation (probably fat capsules), rather periportally. The nuclei remain in the center of hepatocytes in most cells. In TM5 samples (*n* = 7), some hepatocytes had small dose vacuolation of the cytoplasm (probably fat sacs), rather periportally. In the fourth examined sample of the TM5 group, the changes from all three groups were most intensely visible. The nuclei remain in the center of hepatocytes in most cells. In general, all examined samples were free of pathological changes. See Figure 1.

### 3.3. Microbial Colonization in Cecal Chyme

In the 42nd week of hens’ age, a chyme analysis from *caecum* was performed. The results are shown in Table 4. Nevertheless, the table shows that no statistically significant differences were found, there was a decrease in the number of colonies of *E. coli* and other coliform bacteria. The presence of the genus *Salmonella* was also analyzed. All samples were negative from the point of view of the presence of this bacteria.

### 3.4. Microbiological Analysis of Experimental Feeds

Results are shown in Table 5. The presence of *E. coli* and *Salmonella* spp. was not found in mealworm meal. Other monitored species of microorganisms were at a very low level in the raw material. Likewise, the mycotoxin content was well below the limit values.

The microbiological quality of the experimental feed mixtures was monitored for four months. As can be seen from the results in Table 6 and Table 7, there was no deterioration in the microbiological parameters of the diets during the monitoring period. The feed mixtures were also tested for *Salmonella* spp. during the storage period. All tested samples were negative during the storage period.

The most problematic is the number of molds, which could completely degrade the feeds, especially by the production of mycotoxin. The high production of mycotoxins was refuted by subsequent analyses. Stored feed mixtures were tested for mycotoxin contamination at the end of the storage period. It was found approximately 338 µg/kg DON, 0.4 µg/kg OTA, <0.1 µg/kg AFB1 and <0.1 µg/kg AF in experimental feed mixtures (in 92.79% dry matter).

## 4. Discussion

In the present study gut morphology, ilea digesta viscosity and cecal chyme microbiology was evaluated with the inclusion of 2% and 5% of mealworm meal in diets. Biasato et al. [27] fed female broilers 50 g/kg, 100 g/kg or 150 g/kg of *Tenebrio molitor* in the diets. They found no influence of the diets on the gut morphometric indices. Our results of ileum villus height corresponded to their findings. Biasato et al. [27] found out that the abdominal fat weight showed a significantly linear response to increasing the TM meal levels, with a maximum corresponding to the inclusion of 150 g/kg of TM meal. No significant effects related to TM meal utilization were observed for the weight of other relative organs. Biasato et al. [27] also found histopathological alterations in liver: a moderate (control, TM 50 g/kg and TM 100 g/kg = 50% of the broilers; TM 150 g/kg = 30%) to severe (control = 20%; TM 50 g/kg = 30%; TM 100 g/kg = 20%; TM 150 g/kg = 0%) perivascular lymphoid tissue activation. A normal liver was observed in 30% of the control group, 20% (50 g/kg), 30% (100 g/kg) and 70% (150 g/kg) of the animals. In our trial, all examined liver samples were free of pathological changes even after 6 months of laying. This means that the inclusion of mealworm meal did not affect liver health or worsen the condition of villi in the laying hen´s ileum. In another experiment of Biasato et al. [28] it was found statistically significant higher ileum villus height in the group receiving 75 g/kg of TM meal compared to the control group of slow-growing chickens. It was logical to perform the experiment on slow-growing chickens, due to their longer fattening—i.e., longer exposure to the tested feed. With longer exposure to the tested feeds, it can be expected that any changes will take effect. Therefore, it makes sense to test insect products in laying hens or slow-growing chicken diets for an extended period.

In trial [29], defatted black soldier fly larvae meal feeding (BSFLM) had no effect on pancreas, small intestine, and gizzard weight of layers. Feeding this insect product quadratically increased the liver weight. The inclusion of BSFLM reduced empty ceca weight linearly and quadratically compared to the control group of layers. Generally, the high crude fiber content in poultry diets tends to increase the sizes of ceca and small intestine weight [29,30]. Other studies showed that chitin present in insects had a positive effect on the gastrointestinal physiology and metabolism of the Lohmann Brown Classic laying hens [31,32]. Chitin may be a substrate for microbial fermentation and therefore, it could have a positive effect on the microbial balance in the gastrointestinal tract similar to a probiotic [33]. We have not confirmed this phenomenon in our experiment.

It is well documented that many enzymes (like arabinoxylans) may increase digesta viscosity [34], which might lead to a slower passage rate and reduced absorption of nutrients which can lead to worse growth in broilers [35,36]. Moreover, another negative effect of the viscosity is the ability to hold water in the digesta, producing adhesive excreta and increased moisture of the litter [37,38]. Increased intestinal viscosity may change the morphology of the ileal villi [34,39]. Due to a lack of endogenous enzymes that degrade dietary fiber, including soluble non-starch polysaccharides (NSP), the intestinal viscosity increases, which slows down the migration and absorption of nutrients [40]. For example, the ileal digesta viscosity [41] (but measured at a temperature of 25 °C) was lower compared to our results (2.45 vs. 5.43 mPa·s). In our experiment, the ileal viscosity was higher with an increased proportion of TM in the diets. The reason for the increase of the ileal viscosity in our experiment, with a proportion of 5% TM in the diet, may be the presence of chitin, which has similar digestive properties as crude fiber. On the other hand, the crude fiber content was not particularly different in each experimental diet (Table 1). It should be noted that the increased ileal digesta viscosity in TM5 affected neither the length of the villi, nor the length and weight of the ileum and vice versa.

There are no studies to verify the stability of feed mixtures with the proportion of insect products. For this reason, it is difficult to compare our results. However, information about insect microbial flora may be helpful. Generally, the microbial flora of insects is composed of bacteria of different genera: *Staphylococcus, Streptococcus, Bacillus, Proteus, Pseudomonas, Escherichia, Micrococcus, Lactobacillus* and *Acinetobacter* [42,43,44,45]. The largest variations were found in numbers of bacterial endospores, psychrotrophs and fungi. *Salmonella* spp. and *L. monocytogenes* were not detected in any of the fresh mealworm larvae samples [46]. Insect microflora can affect the microbial stability of feeds and it may also affect the gut microbiome of animals. There was a decrease in the number of colonies of *E. coli* and other coliform bacteria (Table 4) in ceca in our trial. This decrease could be due to the antibacterial agents that *Tenebrio molitor* is able to produce. Shin et al. [47] observed in antimicrobial tests that chitosan (produced by alkaline deacetylation of chitin) from the larva of Mealworm Beetle showed about 1–2 mm inhibition zones against four strains of bacteria: *S. aureus, B. cereus, L. monocytogenes*, and *E. coli,* indicating antimicrobial activity. If we focus on the detection of *E. coli*, it is very positive that there were no findings in any sample of the tested feed during the storage period.

In the present study it was also tested the presence of mycotoxins, both in mealworm meal and in the feed mixtures with a proportion of this insect product during storage. Under incorrect storage conditions, fungi may form in the feeds, which may form mycotoxins. According to Commission recommendation 2006/576/EC [48] and Commission regulation 574/2011 [49] a content of 0.1 mg/kg OTA, 5 mg/kg DON, 0.02 mg/kg AFB1 is determined for feed mixtures for poultry (in 88% dry matter—as fed basis). The mycotoxin levels found in our experimental feed mixtures containing mealworm meal are well below the limit values.

While replacing traditional sources of protein with insect-based protein in the layer diet, economic efficiency has to be taken into account. Recently, the prices of insect protein have been several times higher than sources of the traditional protein used in the layer diet. Therefore, it makes sense to keep the proportion of the insect protein at a lower level, which is not significantly affecting the price of the produced feed, but, at the same time, it has the potential to affect positively the production capacity of hens or the health of hens during their productive life. The inclusion of a small percentage of the insect protein can also be used in marketing activity related to selling final products because a part of the customers positively evaluates the replacement of the traditional sources of protein by the insect protein. The impact on the final price has always to be controlled, as it is a crucial indicator for the economic efficiency from the point of view of commercial producers of both feed and laying hens.

By increasing the scale of production, insect farmers will be able to increase the price competitiveness and stability of their products compared with other protein sources. Automation and controlled production systems will significantly help stakeholders to achieve this by making insect production less labor-intensive [50]. To establish the economic impact of adding insects into animal feeding and more cost–benefit analysis will have to be carried out to deeply investigate how these alternative ingredients effectively influence overall production costs. In particular, the offset of the extra costs of novel feeds by the improvement of animal health and performance, as well as the market premium potentially derived from higher welfare products, will have to be considered [51].

In the present study, the mealworm meal inclusion did not affect the morphology of the small intestine, thus suggesting no influence on nutrient metabolization or performance. The full-fat mealworm meal inclusion did not induce histological changes, thus suggesting no negative influence on animal health.

## 5. Conclusions

Two and five percent of mealworm meal were included in layers’ diet. Based on our results, it may be concluded that the proportion of the mealworm meal does not deteriorate the quality of feeds. The mealworm meal does not negatively affect the microbial stability (and production of mycotoxins) in experimental feeds. In the experiment, it was found out that the proportion of 2% and 5% of the mealworm meal in hens’ diet did not affect the length of villi and the microbiome of the caecum. At the same time, the highest digesta viscosity of the ileum was found in the TM5 group, which may indicate a slower passage of the digestion through the digestive tract. Therefore, the inclusion of two and (or) five percent of the mealworm meal in hens’ diet can be recommended.

## Figures and Tables

**Table 1:** Composition and nutrient content of the experimental diets.

Component (%)	TM0	TM2	TM5
Maize	32.60	32.30	31.40
Soybean meal	30.30	27.90	24.10
Wheat	19.19	20.04	22.54
CaCO_3_	7.75	7.75	7.75
Rapeseed oil	4.35	4.20	3.50
Vitamin-mineral premix *	3.00	3.00	3.00
Monocalcium phosphate	2.20	2.20	2.20
Mealworm meal	0.00	2.00	5.00
NaCl	0.30	0.30	0.30
DL-Methionine	0.20	0.20	0.20
NaHCO_3_	0.10	0.10	-
L-Threonine	0.01	0.01	0.01
Analyzed composition (as fed)			
Metabolizable energy (MJ/kg)	11.42	11.48	11.48
Crude protein (%)	18.83	18.13	18.18
Ether extract (%)	5.52	5.97	6.01
Crude fiber (%)	1.79	1.93	1.97
Crude ash (%)	14.50	14.76	14.50
Calcium (%)	3.55	3.70	3.52
Total phosphorus (%)	0.78	0.80	0.79

TM0—0% mealworm meal; TM2—2% mealworm meal; TM5—5% mealworm meal. * Vitamin-mineral premix per kg diet: 0.39 g lysine; 1.35 g methionine; 8.85 g Ca; 2.01 g P; 1.38 g Na; 9.00 mg Cu; 54.00 mg Zn; 60 mg Fe; 72.00 mg Mn; 0.9 mg I; 0.24 mg Se; 9900 IU vitamin A; 3000 IU vitamin D_3_; 15.00 mg vitamin E; 1.2 mg B_1_; 3.6 mg B_2_; 1.62 mg B_6_; 12.00 mg B_12_; 0.09 mg biotin; 0.9 mg folic acid; 12.6 mg niacinamide; 7.5 mg calcium pantothenate; 180 mg choline chloride; 0.3 mg butylhydroxyanisole; 1.5 mg butylhydroxytoluene; 3 mg etoxyquin.

**Table 2:** Relative sizes of individual sections of the digestive tract of hens (per kg of live weight).

	TM0	TM2	TM5	*p* Values
*n*	7	7	7	
	mean ± SEM			
Final live weight (g)	1912 ± 47.58	1978 ± 37.94	1950 ± 52.60	0.612
Crop, weight (g)	3.78 ± 0.40	3.26 ± 0.22	3.72 ± 0.31	0.465
Proventriculus, weight (g)	4.40 ± 0.13	4.81 ± 0.24	4.66 ± 0.19	0.320
Gizzard, weight (g)	12.19 ± 0.55	12.90 ± 0.47	13.04 ± 0.48	0.447
Gizzard, length (mm)	25.34 ± 0.49	25.95 ± 0.30	26.10 ± 0.69	0.557
Gizzard, width (mm)	19.85 ± 0.55 ^a^	21.63 ± 0.43 ^b^	21.45 ± 0.27 ^ab^	0.017
Gizzard, height (mm)	11.01 ± 0.29 ^a^	11.72 ± 0.20 ^ab^	12.42 ± 0.23 ^b^	0.003
Gizzard, muscle height (mm)	7.76 ± 0.37 ^b^	6.37 ± 0.43 ^a^	6.88 ± 0.22 ^ab^	0.038
Duodenum, weight (g)	4.30 ± 0.26	4.47 ± 0.32	4.39 ± 0.24	0.930
Duodenum, length (mm)	154.68 ± 4.52	163.28 ± 6.06	156.45 ± 7.10	0.277
Jejunum, weight (g)	8.30 ± 0.51	9.72 ± 0.61	9.16 ± 0.53	0.384
Jejunum, length (mm)	312.40 ± 17.00	323.60 ± 12.90	301.90 ± 11.90	0.749
Ileum, weight (g)	11.72 ± 0.91	10.86 ± 0.62	9.56 ± 0.64	0.902
Ileum, length (mm)	233.30 ± 24.90	233.30 ± 11.30	222.70 ± 10.30	0.573
Ceca, weight (g)	4.67 ± 0.20	5.03 ± 0.27	4.65 ± 0.21	0.214
Ceca, length (mm)	78.7 ± 03.2	77.5 ± 02.0	77.2 ± 04.2	0.562
Colon, weight (g)	3.13 ± 0.17	3.21 ± 0.17	2.84 ± 0.14	0.140
Colon, length (mm)	73.90 ± 3.50 ^b^	64.0 ± 2.00 ^ab^	59.90 ± 03.10 ^a^	0.876
Liver, weight (g)	17.12 ± 0.38	17.41 ± 0.90	17.45 ± 0.62	0.269
Spleen, weight (g)	0.97 ± 0.06	1.19 ± 0.15	1.11 ± 0.04	0.277
Heart, weight (g)	3.62 ± 0.13	3.72 ± 0.20	3.41 ± 0.12	0.431
Abdominal fat, weight (g)	48.44 ± 3.49	50.94 ± 4.92	44.98 ± 7.41	0.936

TM0—0% mealworm meal; TM2—2% mealworm meal; TM5—5% mealworm meal. SEM—standard error of the mean. ^a,b^—different letters in one column mean statistical differences (*p* < 0.05).

**Table 3:** Ileum villus height and ileum digesta viscosity.

	*n*	Villus Height (µm)	Digesta Viscosity (mPa·s)
		mean ± SEM	
**TM0**	7	571.96 ± 26.63	4.49 ± 0.22 ^a^
**TM2**	7	602.32 ± 35.37	4.74 ± 0.15 ^ab^
**TM5**	7	591.08 ± 28.51	5.43 ± 0.30 ^b^
*p* values		0.778	0.026

TM0—0% mealworm meal; TM2—2% mealworm meal; TM5—5% mealworm meal. SEM—standard error of the mean. ^a,b^—different letters in one column mean statistical differences (*p* < 0.05).

**Table 4:** Ceca microbiota (log CFU/g).

	*n*	*E. coli*	Other Coliform Bacteria	*Clostridium perfringens*
		mean ± SEM		
**TM0**	7	7.41 ± 6.98	7.17 ± 7.05	0.70 ± 0.56
**TM2**	7	6.72 ± 6.38	4.04 ± 3.85	1.29 ± 1.27
**TM5**	7	6.89 ± 6.55	2.81 ± 2.81	2.16 ± 2.01
*p* Values		0.056	0.203	0.219

TM0—0% mealworm meal; TM2—2% mealworm meal; TM5—5% mealworm meal. SEM—standard error of the mean. CFU—colony-forming unit. There were not found statistical differences (*p* > 0.05).

**Table 5:** Mealworm meal microbial (log CFU) and mycotoxins (mg/kg) analyses.

Other Coliform Bacteria	Enterococci	*Clostridium perfringens*	TPC	LAB	Yeasts	*S. aureus*	Molds	DON	OTA	AFB1	AF
2.08	1.62	0.23	3.13	0.32	0.23	1.80	2.30	<40.0	1.7	<0.1	<0.1

Mycotoxin content was analyzed in 94.3% of dry matter. TPC—total plate count; LAB—lactic acid bacteria; CFU—colony-forming unit. OTA—ochratoxin A, AF—aflatoxins, AFB1—aflatoxin B1, DON—deoxynivalenol.

**Table 6:** Feed microbial analysis at the beginning of the storage period (log CFU).

	*E. coli*	Other Coliform Bacteria	Enterococci	*Clostridium perfringens*	TPC	LAB	Yeasts	Molds	*S. aureus*
**TM0**	ND	5.20	2.36	ND	5.63	2.74	2.64	3.64	2.32
**TM2**	ND	5.27	2.24	ND	5.48	2.63	1.62	3.62	1.60
**TM5**	ND	5.14	2.13	ND	5.84	2.48	2.47	3.60	1.68

TM0—0% mealworm meal; TM2—2% mealworm meal; TM5—5% mealworm meal. TPC—total plate count; LAB—lactic acid bacteria; ND—non detected; CFU—colony-forming unit.

**Table 7:** Feed microbial analysis at the end of the storage period (log CFU).

	*E. coli*	Other Coliform Bacteria	Enterococci	*Clostridium perfringens*	TPC	LAB	Yeasts	Molds	*S. aureus*
**TM0**	ND	4.99	2.42	ND	5.34	ND	2.40	3.54	2.70
**TM2**	ND	5.02	2.45	ND	5.45	ND	2.40	3.35	2.50
**TM5**	ND	5.11	2.57	ND	5.54	ND	2.40	3.60	2.16

TM0—0% mealworm meal; TM2—2% mealworm meal; TM5—5% mealworm meal. TPC—total plate count; LAB—lactic acid bacteria; ND—non detected; CFU—colony-forming unit.

## Data Availability

The data presented in this study are available on request from the corresponding author. The data are not publicly available due to ongoing patent proceedings.
